# Identification and functional analysis of growth rate associated long non-coding RNAs in *Komagataella phaffii*

**DOI:** 10.1016/j.csbj.2025.04.028

**Published:** 2025-04-22

**Authors:** Benjamin Luke Coltman, Krishna Motheramgari, Nadine Tatto, Brigitte Gasser

**Affiliations:** a CD-Laboratory for Growth-decoupled Protein Production in Yeast at Department of Biotechnology, BOKU University, Vienna, Austria; b Department of Biotechnology and Food Science, Institute of Microbiology and Microbial Biotechnology, BOKU University, Vienna, Austria; c Division of Microbial Ecology, Centre for Microbiology and Environmental Systems Science, University of Vienna, Vienna, Austria; d Austrian Centre of Industrial Biotechnology (acib), Vienna, Austria; e Vienna Biocenter Core Facilities GmbH (VBCF), Next Generation Sequencing, Vienna, Austria

**Keywords:** lncRNA, *Komagataella phaffii*, *Pichia pastoris*, Near-zero growth, Retentostat

## Abstract

Long non-coding RNAs (lncRNAs) are a regulatory feature that have been reported to operate on both transcriptional and translational levels. With DNA-based prediction still limited, lncRNAs are most reliably identified through genome-guided mapping of RNA-Seq data. Reports of lncRNAs in yeast have been increasing in recent years and changes in their expression levels have often been associated with stressful conditions. As the transition to near zero-growth conditions likely imposes stress, we used RNA-Seq data from the non-conventional, biotechnologically established yeast *Komagataella phaffii,* cultivated in glucose-limited retentostats, to identify the expression of lncRNAs. Using an adapted bioinformatics pipeline, we identified 168 mostly novel lncRNAs from the *K. phaffii* retentostat RNA-Seq data, 36 of which demonstrate likely growth-associated expression changes. lncRNA expression levels were associated to that of possible interaction partners, in both *cis* and *trans*, suggesting potential roles in regulatory adaptations. Our analysis indicates that lncRNAs likely contribute to how *K. phaffii* responds to changing environmental conditions, as exemplified here by the adaptation to extremely slow growth.

## Graphical abstract

**Figure:**
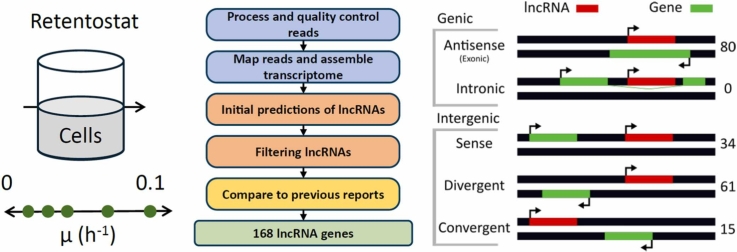

## 1 Introduction

Long non-coding RNAs (lncRNAs) are increasingly implicated in a wide range of cellular processes. lncRNAs are a class of non-coding RNAs that are over 200 nucleotides long and have been recognised for their critical regulatory functions in gene expression, both in proximity (*cis*) and at distant sites (*trans*) relative to their genomic location. These functions include the recruitment of epigenetic modifying complexes and possible interactions with other DNA, RNA, protein, and small molecules [1]. While advances in sequencing and transcriptome analysis have facilitated the identification of lncRNAs, understanding their biological roles remains a considerable challenge. Large-scale projects like ENCODE and GENCODE have increased the number of annotated functional genomic elements in humans and mice, including lncRNAs [2], [3]. Their annotation and functions have also been reported in lower eukaryotes, including yeast [4], [5], [6].

Yeasts possess a relatively simpler genomic landscape compared to the more complex mammalian eukaryotes, which could potentially ease the study of lncRNAs, allowing for clearer insights into their functional mechanisms. However, lncRNA sequences are typically less well conserved compared to that of protein-coding genes. Several studies have looked at lncRNAs in the model yeast species *Saccharomyces cerevisiae* and *Schizosaccharomyces pombe*, linking lncRNAs to diverse functions such as the glycolytic to gluconeogenic switch and flocculation [6], [7], [8]. lncRNAs have also been predicted in some non-conventional yeasts, including the popular recombinant protein production platform, *Komagataella phaffii*. Since the first publicly available genome annotations of *K. phaffii* and *Komagataella pastoris* (both at the time still commonly referred to as *Pichia pastoris*) were reported in 2009 [9], [10], genome sequences and annotations of different strains have been added, including a few rounds of manual annotation [11], [12], [13], [14]. Non-coding annotations were also included in the annotation update of the strain CBS7435 by Valli and coworkers [14] and Sturmberger and coworkers [13], which included annotations of tRNAs, rRNAs and snoRNAs.

Despite the fairly comprehensive annotations of *K. phaffii* coding genes, lncRNAs are still underexplored in *K. phaffii*, with two studies discussing their identification [15], [16]. Using the originally published genome sequence of the commercial strain GS115 [9] and published RNAseq data [17], Schneider and coworkers [15] annotated 56 possible lncRNAs in the GS115 genome sequence. However, their analysis was limited by the library preparation method used to generate the RNAseq data (polyA enrichment of transcripts and a stranded preparation), particularly affecting the annotation of antisense lncRNAs. Sun and coworkers [16] annotated 208 lncRNAs in a *K. phaffii* strain producing a recombinant protein (phospholipase PLA2) under the control of a methanol inducible promoter. By analysing the transcriptome at multiple points during a multi-stage fed-batch cultivation (glycerol batch, glycerol fed-batch, methanol fed-batch), they demonstrated a carbon-source dependent regulatory response and suggested possible interactions of the identified lncRNAs with other genes. However, comprehensive details of the lncRNAs were not reported and subsequently, information about lncRNAs in *K. phaffii* remained largely unavailable.

lncRNAs have been implicated in a number of cellular processes, particularly in the responses to stress [6], [18], [19], [20]. We have recently investigated the metabolic shifts [21] and transcriptional response [22] of *K. phaffii* during glucose-limited retentostat cultivations, where the cells transition to near-zero growth rates. As the adaptation of cells to extremely slow (near-zero) growth conditions clearly represents such a stressful condition, we hypothesised that lncRNAs might play a role. Using the RNASeq data obtained from the retentostat cultivations of *K. phaffii* at several different growth rates [22], we annotated lncRNAs, investigate their growth rate dependent expression levels and generated functional hypothesis for their modes of action. The findings from our analysis bridge prior metabolic [21] and transcriptional [22] insights with potential genomic regulation, offering a complementary perspective on the adaptation of *K. phaffii* to near-zero growth conditions.

## 2 Result

### 2.1 Identification of long non-coding RNAs

We annotated lncRNAs in *K. phaffii* using RNAseq data from glucose-limited cultivations of a recombinant vHH secreting strain across a more than 200-fold range of growth rates (ranging from 0.00047 to 0.1 h^−1^). The RNAseq data was previously generated in a study demonstrating the feasibility of recombinant protein secretion at extremely slow growth rates [22]. The study generated 24 RNAseq libraries, each sequenced to a depth of more than 24 million paired-end 150 bp reads, distributed across 8 sampling points (NCBI SRA, PRJNA1013119). One sampling point was from a steady state chemostat at a growth rate of 0.1 h^−1^ (C0.1), the other 7 samples were from retentostat cultivations, initiated from steady state chemostats at a growth rate of 0.025 h^−1^. The retentostats were initiated after two weeks (short chemostat, SC) or three weeks (long chemostat, LC) of chemostat cultivation. Unexpectedly this led to different profiles in the retentostat cultivation, and differences in cell morphology [22]. These cultivations were sampled: prior to retentostat initiation (0.025), after ∼6 days (R3), after ∼14 days (R6, only for SC) and after ∼28 days (R10). The samples from the LC cultivations were utilised for lncRNA annotation but are not further investigated in this analysis. In the SC cultivations, regression models of the retentostat cultivation predicted growth rates of ∼0.0022 h^−1^, ∼0.0010 h^−1^ and ∼0.0005 h^−1^ at sampling points R3, R6 and R10, respectively [21], [22].

**Fig. 1:**
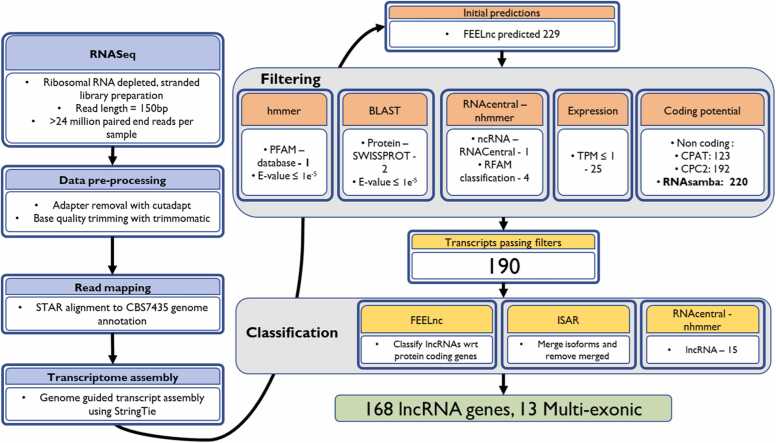
Overview of the lncRNA identification pipeline used for *K. phaffii* lncRNA annotation.

A lncRNA annotation pipeline was developed for *K. phaffii*, similar to that reported for the annotation of lncRNAs in Chinese hamster ovary (CHO) cells [23]. In short, the pipeline consisted of the quality control of sequencing reads, mapping to the genome, transcriptome assembly, lncRNA classification and extensive filtering to remove coding transcripts (Fig. 1). The main outcome was the identification of 168 lncRNAs, 13 of which were multi-exonic.

Reads were trimmed to remove low-quality bases and contaminating adapter sequences, prior to mapping to the CBS7435 genome using STAR [24]. The strain used in retentostat cultivations was generated in the CBS2612 background; however, due to the high similarity between the two strains (∼4 SNPs) and comprehensive, manually refined annotation of CBS7435, the CBS7435 genome annotation was used [11], [14]. After mapping, the transcriptome was assembled using Stringtie [25], yielding 6097 transcripts (843 multi-exonic) in 5818 loci, which represent an additional 437 transcripts (252 multi-exonic) and 181 loci compared to the reference annotation used. Of the newly annotated transcripts, those less than 200 nucleotides in length and/or overlapping existing annotations in-sense (on the same strand) were discarded, before initial classification of the resulting 229 transcripts with the lncRNA discovery algorithm FEELnc [26].

All of these transcripts were predicted to be lncRNAs when using FEELnc’s default method of randomly shuffling mRNA. When using a balanced coding/non-coding integrated dataset from CPPred as the training data for the FEELnc classifier, a similar result was obtained with only 7 of the transcripts predicted to be mRNA [27]. We chose to continue with the 229 transcripts (all of the transcripts > 200 nt) predicted as non-coding by the default method of FEELnc and further investigated these transcripts with additional coding-prediction tools including RNAsamba, CPAT and CPC2 [28], [29], [30]. These three tools were assessed by comparing their abilities to classify the coding/non-coding sequences annotated in the CBS7435 genome (Supplementary Table 1). FEELnc was not benchmarked as the same data used for the random shuffling approach would be used in its evaluation. The pre-trained RNAsamba model achieved the highest F1 score in classifying pre-existing annotations, while the RNAsamba model trained with the balanced CPPred dataset achieved the best balanced accuracy. In terms of F1 score and balanced accuracy, CPAT and CPC2 performed worse than RNAsamba (Supplementary Table 1) and we therefore decided not to consider these two prediction tools for filtering out lncRNAs according to coding-potential. The 229 transcripts predicted as lncRNAs by FEELnc were classified with an ensemble RNAsamba model, predicting that 220 of the transcripts were non-coding. This ensemble model consisted of both the pre-trained model and an additional RNAsamba model trained with the balanced CPPred dataset extended with the pre-existing CBS7435 annotations. CPAT and CPC2, whose results were not considered for filtering out transcripts, predicted that 123 and 192 of the 229 transcripts were lncRNAs, respectively.

Potential protein domains were predicted in open reading frames within the lncRNAs. For this, open reading frames longer than 100 amino acids were translated from all transcripts using TransDecoder [31] and for each lncRNA, the longest open reading frame was retained. BLAST hits of the translated sequences were inspected against the SWISSPROT database, whilst protein domains were annotated using hmmer against PFAM domain models, yielding 1 and 2 hits, respectively [32], [33].

The nucleotide sequences were queried against RNAcentral, which included querying the RFAM database for domains [34], [35]. RFAM querying suggested four of the transcripts contained ncRNA domains (E-value < 1e-5). Seven hits annotated as miscRNA were returned from querying RNAcentral, with all but one derived from a different annotation of *K. phaffii* [13]. The single hit that was not derived from *K. phaffii* was also annotated as a tRNA by the RFAM query and was subsequently excluded in the later intersection of filters. One hit was annotated as a snoRNA in the GS115 annotation and was also confirmed in the RFAM query. The MSTRG.1715 sequence returned two hits to features annotated as a 35S promoter and 35S pre-rRNA, yet querying RFAM did not confirm the presence of any feature in the MSTRG.1715 sequence. We investigated this further and found that in our more recent annotation the sequences corresponding to the returned hits are now annotated as multiple features, and that the gene neighbouring MTRG.1715, PP7435_Chr1–1624, is instead annotated as a 35S pre-ribosomal RNA. Therefore, we chose not to exclude MSTRG.1715 based on these hits. The additional three hits contained no further information or sub-classification than miscRNA, which did not exclude them from being lncRNAs. Subsequently, they were not excluded based on this filter. The lncRNAs were queried against the RFAM lncRNA models but no hits were found. The annotation resulting from Stringtie was used by Salmon for selective alignment of the reads and quantification of the transcript abundance [36]. lncRNAs were discarded if their expression levels were not greater than 1 TPM in at least one sample.

The intersection of all filtering steps yielded 190 unique transcripts from 168 genes, 13 of which were multi-exonic (Supplementary File 1). Multiple transcripts were annotated for five of the lncRNA genes. For MSTRG.4363 and MSTRG.4376, 10 or more isoforms were annotated and merged via Stringtie, although this likely reflects the repetitive nature of the region in which they were annotated. In further analysis, expression level was only considered on a gene wise level.

The lncRNAs were classified with respect to protein-coding genes and a less conservative querying against RNAcentral (query coverage > 50 %, E-Value < 1e-2). Of the 168 genes, 12 of them returned hits from RNAcentral, however, the median identity and query coverage of all hits was low, 53 % and 58 %, respectively. RNAs annotated as lncRNAs or miscRNAs accounted for 73 % and 21 % of returned hits, respectively. Of these lncRNAs, 30 % of the hits were to lncRNAs from *S. pombe* , however, only four lncRNAs accounted for these hits. The lncRNA MSTRG.3832, which is antisense to *PRP2*, had 17 hits to lncRNAs in a range of species, including to a lncRNA in *S. pombe* that is antisense to *CDC28*. Interestingly, a lncRNA antisense to *PRP2* has also been reported in *S. pombe* but was not returned as a hit to MSTRG.3832. MSTRG.3443, antisense to *AGP2–2*, had a hit to 4 different lncRNAs in *S. pombe,* yet in *S. pombe* none of these 4 lncRNAs were antisense to *AGP2–2*, with two of them antisense to uncharacterised coding sequences. MSTRG.2029, antisense to *CAR1,* returned a hit to an *S. pombe* lncRNA which is also antisense to *CAR1*. MSTRG.618, antisense to PP7435_Chr1–0580, returned a hit to an *S. pombe* lncRNA that is antisense to *VSB1*; however, there was no sequence similarity between *VSB1* and PP7435_Chr1–0580. The rest of the lncRNA hits were distributed across various species mainly from the plant kingdom.

**Fig. 2:**
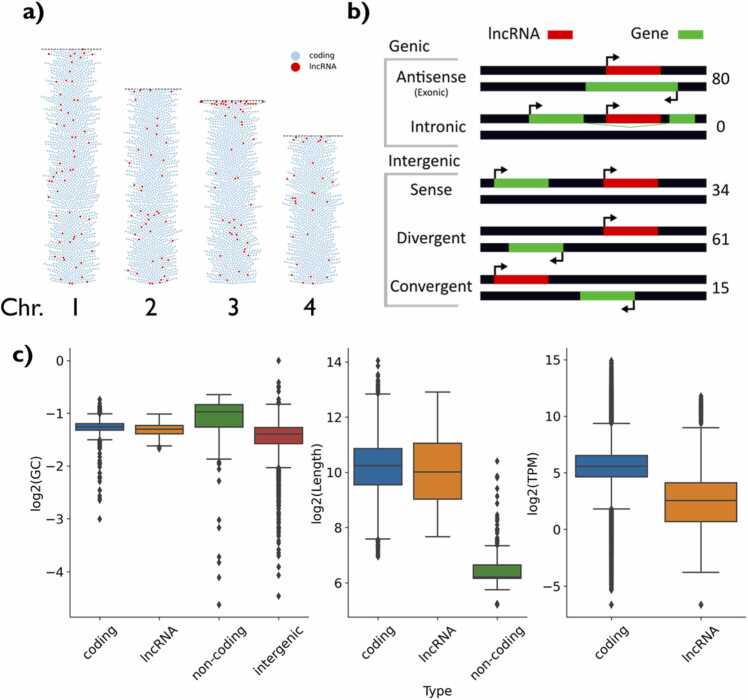
lncRNA characteristics. a) Swarm plot representation of the location of lncRNAs on the chromosomes. Markers represent genes and are coloured according to whether the gene is coding or a lncRNA. b) lncRNAs are categorised with respect to the nearest gene. Counts for the broad categories are shown. c) Molecular properties of identified lncRNAs in comparison to coding genes, non-coding genes or intergenic regions, where applicable. Comparison of left) GC content, centre) transcript length and right) expression level.

The identified lncRNAs are distributed across the genome, but with several identified at sub-telomeric locations (Fig. 2a). Of these 190 lncRNA transcripts: 80 were antisense, exonic to a protein-coding gene; 61 divergent; and the rest various intergenic types (Fig. 2b) (15 convergent, 12 downstream sense, and 22 upstream sense). Several characteristics of the lncRNAs were compared to that of annotated protein-coding genes, non-coding genes (ncRNAs such as tRNAs, rRNAs, and snoRNAs) and intergenic regions (containing no annotations). The identified lncRNAs have a similar GC content and length to protein-coding genes but were expressed at lower levels (Fig. 2c). In comparison to already annotated ncRNAs, they have a lower GC content and are longer, although this is affected by the 200 nt cutoff for annotation as a lncRNA.

The transcript level expression estimates from Salmon were merged to a gene-level and used in further analysis [36]. Next, it was investigated whether the lncRNAs demonstrate a growth rate dependent change in expression level.

### 2.2 lncRNA expression is growth rate dependent

**Fig. 3:**
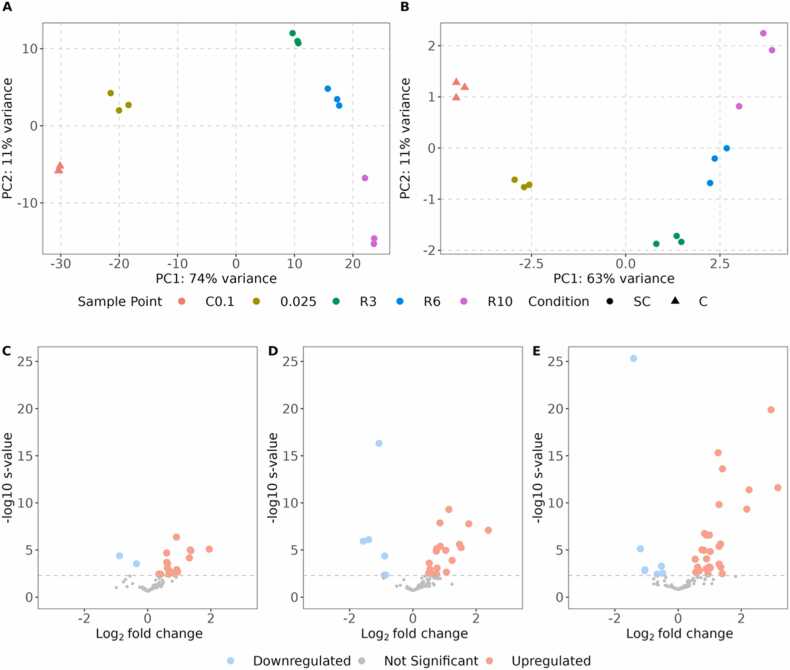
Principal component analysis (PCA) of RNAseq data, showing the first two dimensions based on A) all gene counts and B) lncRNA counts. Marker shapes indicate condition (triangle: chemostat C; circle: retentostat with short chemostat phase SC) and marker colours indicate sampling points. C samples include a steady-state chemostat at 0.1 h^−1^ (C0.1). SC samples include a chemostat at 0.025 h^−1^ prior to retentostat initiation (0.025) and the retentostat cultivation sampled ∼6 (R3, ∼0.0022 h^−1^), ∼14 (R6, ∼0.0010 h^−1^), and ∼28 days (R10, ∼0.0005 h^−1^) after initiation. C-E) Expression profiles of lncRNA genes are shown in volcano plots, representing differential expression within the SC condition between the initial 0.025 sample and the C) R3, D) R6, and E) R10 sampling points. In the volcano plot, dots coloured in light red or light blue denote statistically significant up- or downregulation of lncRNAs, respectively (s-value < 0.005).

Principal component analysis suggested that growth rate was the main source of variability between all RNAseq libraries when looking at gene-level counts of all genes and a subset of just the newly annotated lncRNAs (Fig. 3a & b). The first principal component accounted for 74 % of variance for all genes and 4 % for the lncRNA subset (Fig. 3a). The first two components accounted for 85 % of the variance for all transcripts and 5 % for the lncRNA subset (Fig. 3b). Hierarchical clustering of the samples, using the counts of all genes, demonstrated a similar clustering according to growth rate (Supplementary Figure 1a). When clustered on the counts of all lncRNAs, the differences were similarly clear, suggesting that growth rate is also a source of reasonably large variability for lncRNA expression (Supplementary Figure 1b). In both cases, the samples from the highest growth rates (C0.1 and 0.025) clustered together, whilst the other 3 sampling points were also closely clustered.

36 of the 168 lncRNA genes demonstrated a significant growth rate dependent expression. This was determined from a likelihood ratio test comparing a model accounting for the different sampling points (i.e. *growth rates*) and a reduced model without these variables. Pairwise differential expression analysis was also performed within the SC retentostat samples, with the three sampling points (R3 ∼0.0022 h^−1^, R6 ∼0.0010 h^−1^ and R10 ∼0.0005 h^−1^) compared to the chemostat cultivation at 0.025 h^−1^. Here, 40 of the lncRNAs were significantly differentially expressed (s-value < 0.005) in at least one pair-wise comparison, with 12 lncRNAs upregulated and 2 downregulated in all three comparisons (Supplementary File 2). When cells were approaching slower growth rates, more lncRNAs were differentially expressed compared to 0.025 h^−1^, with 2 lncRNAs downregulated and 17 upregulated at R3 (∼0.0022 h^−1^, Fig. 3c), and 6 downregulated and 18 upregulated at R6 (∼0.0010 h^−1^, Fig. 3d). At the end of the retentostat (R10, ∼0.0005 h^−1^), the abundance of 35 lncRNAs differed significantly, with 7 lncRNAs downregulated and 29 upregulated (Fig. 3e). Of the 40 lncRNAs differentially expressed in at least one comparison, 18 were antisense to a coding gene, 12 divergent, 5 downstream antisense, 3 upstream sense and 2 downstream sense (Table 1).

**Table 1:** Overview of the 40 lncRNAs that are differentially expressed in at least one pairwise comparison of the respective sample point with the 0.025 sample. Cells are coloured either blue or red if they are significantly up- or down-regulated (S-value < 0.005). For extended details refer to Supplementary File 2.

In the comparison between 0.025 and R10 (0.025 h^−1^ and ∼0.0005 h^−1^), the slowest and fastest growth rates within the retentostat, the three most strongly differentially up-regulated lncRNAs (log_2_FC between 2.2 and 3.2) were closest to *AGP2–2 (*Supplementary Figure 2*)*, and two hypothetical genes (PP7435_Chr1–1395, PP7435_Chr3–1809). The three most strongly downregulated lncRNAs (a log_2_FC between −1.48 and −1.05) were closest to *YHP1* and two hypothetical proteins (PP7435_Chr1–1039, PP7435_Chr1–1607). Other lncRNAs that were differentially expressed in this comparison were antisense to genes including *LIH1–2* (Supplementary Figure 3), *ADH600*, *AOX2*, *SPO11* and *SAP1* (Supplementary File 2).

The mode of action of lncRNAs can be broadly categorised into *cis* and *trans* acting, regulating other genes in proximity or at distant sites relative to their genomic location, respectively. To better understand lncRNA function, we investigated their predicted interactions with protein-coding genes and their patterns in expression levels.

### 2.3 Predictions of lncRNA function

To identify groups of genes with coordinated expression patterns that may reflect shared biological responses or functional associations, we clustered genes based on their temporal profiles. Only genes with significant growth rate dependent expression (includes 36 of the 168 lncRNAs), as determined by a likelihood ratio test (see Methods) were included in the clustering. While co-expression does not imply direct regulatory interaction, it can highlight genes that respond similarly to physiological changes and that may participate in related processes. The gene counts generated by Salmon were variance-stabilised and normalised for library-size. Then, for each gene, these normalised counts were z-score normalised across sampling points and soft-clustered with Mfuzz based on their expression profiles [37]. The genes clustered into four distinct groups with lncRNAs present in each of the identified clusters (Supplementary Figure 4). The clusters were enriched in various biological processes, including ribosome and peptide biosynthesis, cell cycle regulation, and membrane-related processes such as mitochondrial biogenesis (Supplementary File 3). An additional alternative clustering method was employed that considered both expression profile and level, using a variational approach to fit a multivariate Poisson-lognormal (MPLN) model to the expression count data [38], [39]. Seven clusters were identified in this approach, again linking lncRNAs to genes involved in a wide range of biological processes (Supplementary Figure 5, Supplementary Figure 6 and Supplementary File 4).

Based on similar expression profiles and levels, lncRNAs clustered with genes that are associated with a wide array of various cellular processes. However, clear mechanisms of interaction provide stronger evidence for a proposed function than association analyses such as clustering and correlation. Numerous investigations have identified co-expression and localisation in promoter regions as key mechanisms for *cis*-acting lncRNAs. We observed such effects from representative coverage plots of the most strongly up- and down-regulated antisense lncRNAs, MSTRG.3443 (Supplementary Figure 2) and MSTRG.1680 (Supplementary Figure 3), respectively. MSTRG.3443 is antisense to *AGP2–2* and their expression levels are negatively correlated (r = -0.71, p = 0.003, n=15) (Supplementary Figure 2). MSTRG.1680 is antisense to PP7435_Chr1–1564 but divergent to PP7435_Chr1–1565 (Supplementary Figure 3); MSTRG.1680 is negatively correlated to PP7435_Chr1–1564 (*LIH1–2*, r = -0.72, p = 0.002, n = 15) but strongly positively correlated to PP7435_Chr1–1565 (Hypothetical protein, r = 0.92, p < 0.001, n = 15).

Trans-acting lncRNAs may affect other transcripts in several ways, one being the formation of RNA-DNA triplexes in regulatory regions, which affects gene expression. Whilst not extensively studied in non-mammalian cells, recent research further suggests RNA–DNA triplex formation in regulatory regions as a potential interaction method between lncRNAs and protein-coding genes [6], [40], [41]. Using triplexator [42], triplex forming oligos (TFOs) were annotated in the *K. phaffii* lncRNAs and their complementary triplex forming target sites (TTSs) annotated in the CBS7435 genome. 4097 potential interactions (TTS/TFO pairs) were predicted, with 131 of the lncRNAs containing TFOs predicted to form triplexes with 1660 unique TTSs. Many lncRNAs were predicted to be involved in multiple interactions (mean = 31.3, median = 11) with MSTRG.881, MSTRG.312, MSTRG.4427 and MSTRG.5198 predicted to be involved in 378, 372, 265, and 142 interactions, respectively (Supplementary File 5). However, all four of these lncRNAs were expressed at very low levels (maximum TPM of 4, 3, 16 and 6, respectively) and not differentially expressed in the analysed conditions.

First, we looked at the interactions of lncRNAs with TTSs across the whole gene and promoter region. We defined the promoter region as 1500 bp upstream and 250 bp downstream of the transcript start site, chosen to capture interactions close to the transcript start site and to cover the majority of promoter lengths observed in the related yeast *S. cerevisiae* [43]. 3256 potential interactions with 995 genes were predicted. Of these 995 genes, most had a small number of interactions (mean 2.4, median 1), but 52 genes were involved in more than 10 interactions. PP7435_Chr1–1199, a regulator of mannosylphosphate transferase, was involved in 152 interactions Additionally, a number of genes predicted to interact with more than 20 lncRNAs are involved in functions such as ribosome biogenesis, rRNA processing, and protein translation, with many components of snoRNP complexes, preribosomal particles, and the RNA polymerase machinery. Several proteins are chaperones, helicases, or enzymes like mannosyltransferases and ATPases, playing critical roles in RNA modification, cellular stress responses, and vacuole- or Golgi-associated functions. However, despite triplexes being predicted across the whole gene, it is likely that regulatory effects are confined to the promoter region. Therefore, we investigated where the TTS of the interacting pairs were annotated.

The interactions, based on the TTS of the pair, were assigned exclusively to a genomic context using a hierarchical classification scheme (promoter > exon > intron > intergenic). If a TTS overlapped with both a promoter and exon, then the interaction was only classed as promoter. The majority of interactions were classified as promoter exclusive (n = 2048, 997 unique TTSs, 1175 promoters contain a TTS), followed by exon-exclusive (n = 1120, 616 unique TTSs, 504 exons contain a TTS), intergenic-exclusive (n = 65, 47 unique TTSs) and intron-exclusive (n = 3, 3 unique TTSs, 3 introns contain a TTS). For reference, when classifying the genome in the same approach, promoter regions account for 52 % of the genome, intergenic regions 47 %, exon 0.7 % and introns 0.3 %. 79 lncRNAs were predicted to interact with the promoter regions of more than 5 coding genes. For each of the lncRNAs predicted to interact with more than 5 coding genes, an enrichment analysis was performed on their partners. The partners of 16 of the lncRNAs returned potential enrichments of GO terms including amide and peptide biosynthesis, cell communication, stimulus response and lipid metabolism and KEGG terms relating to fatty acid and lipid biosynthesis (Supplementary File 5).

**Fig. 4:**
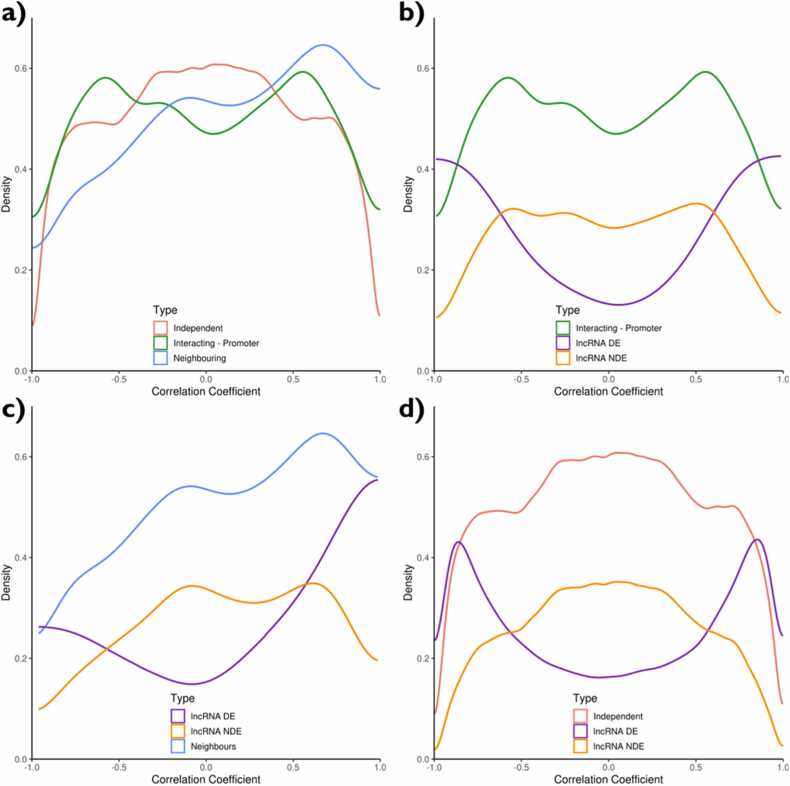
Correlation coefficients of lncRNA and coding genes. Density plots of the correlation coefficient of: a) lncRNAs and coding genes. Different coloured lines represent whether the distribution includes the correlation coefficient of lncRNAs and coding genes within 2 kb of each other (Neighbouring, blue), lncRNAs and coding genes predicted to interact with each other via triplexes in the promoter region (Interacting - Promoter, green) or lncRNAs and coding genes that are independent of each other (independent, red); b) interacting partners, c) neighbouring partners, and d) independent lncRNAs and coding genes, in all cases subset according to whether the lncRNA is growth rate associated (DE, purple) or not (NDE, orange). Growth rate association was determined according to a log ratio test between a full or reduced model and an adjusted p-value < 0.0005.

To determine whether *cis* or *trans* interactions of lncRNAs affected the expression of protein-coding genes, we determined the correlation coefficient between lncRNA and coding gene expression levels and labelled all lncRNA and coding gene pairs according to their potential interactions. If the pairs were within 2 kb of each other, then they were labelled as “Neighbouring” (n = 551); if a TTS in the promoter of a coding gene was linked to a TFO in a lncRNA, then the pair were labelled as “Interacting – Promoter” (n = 2453) and if a coding gene and lncRNA were not neighbouring or predicted to interact then they were labelled as “Independent” (n = 938,251) (Supplementary File 5). The distribution of correlation coefficients for “Neighbouring” and “Interacting – Promoter” pairs differed to each other and to the “Independent” pairs (Fig. 4a). The correlation coefficient of neighbouring lncRNA and protein-coding genes was positively shifted, whilst a bimodal distribution was observed for those lncRNAs and protein-coding genes interacting via triplexes (Fig. 4a). Interacting partners were subset according to whether the lncRNA was differentially expressed or not (determined via a log-ratio test, see Methods). The distinct bimodal distribution of the differentially expressed lncRNAs demonstrates that differentially expressed lncRNAs tend to positively or negatively correlate with their interacting coding genes (Fig. 4b). This might reflect a regulatory role of lncRNAs in *trans* mediated by triplex interactions, with differentially expressed lncRNAs actively suppressing or activating their interacting partners in response to the changing conditions. This was also evidenced in the correlation of the expression patterns of the interacting pairs (Supplementary Figure 7). Most pairs exhibited positive or negative correlation, with the expression patterns shifting at a similar time for both the lncRNAs and protein-coding genes. Typically, this shift occurred between the 0.025 and R3 sampling point (0.025 h^−1^ and ∼0.0022 h^−1^, respectively), which represented the greatest relative change in growth rate (Supplementary Figure 7). However, a similar bimodal distribution was also observed for the correlation coefficient of growth rate associated lncRNAs and other independent protein-coding genes (Fig. 4d). Neighbouring partners were also subset according to whether the lncRNA was differentially expressed or not, and demonstrate that differentially expressed lncRNAs are more likely to be positively correlated to their coding gene partners (Fig. 4c). This positively shifted distribution potentially indicates regulatory co-activation or shared functional pathways of the lncRNAs and their neighbouring genes. However, this could equally be due to a local chromatin structure affecting the expression of both transcripts and not necessarily mediated by the lncRNAs themselves. Comparison of the expression levels of neighbouring pairs of lncRNAs and genes demonstrated that the largest transitions again occurred between 0.025 and R3 sampling point (0.025 h^−1^ and ∼0.0022 h^−1^, respectively), with both lncRNAs and genes showing a similar pattern (Supplementary Figure 8).

We also investigated the correlation coefficient of lncRNA and coding genes where the TTS of the interaction occurs in the exon (“Interacting – Exon”, n = 1009) as well as the whole gene and promoter region (“Interacting – Gene + Promoter”, n = 3465). If the lncRNA of the pair was growth rate associated, then a bimodal distribution was observed for all three “Interacting” classes (Supplementary Figure 9a). Independent of the lncRNA being growth rate associated, a bimodal distribution was observed for “Interacting – Exon” pairs; however, a greater separation was observed for the pairs involving growth rate associated lncRNAs (Supplementary Figure 9b). Based on these observations, triplex interactions both in and outside of the promoter region may impact expression levels and may warrant further investigation.

## 3 Discussion

An increasing number of reports have highlighted the varied, important roles of lncRNAs across a range of eukaryotes [2], [44], [45], [46], including in model yeast species such as *S. cerevisiae* and *S. pombe* [6], [7], [8], [18], [19], [47], [48], [49], [50]*,* but also in the non-conventional yeast *K. phaffii* [15], [16]. It was envisaged that lncRNAs would be differentially expressed in the transition to extremely slow growth rates and may play important roles in the adaptive mechanisms of *K. phaffii* to these stressful conditions. Using RNAseq data from a ∼250-fold range of growth rates [22], we annotated over 150 lncRNAs, observed a general growth rate dependent expression for 36 of them and found evidence for their potential functions both *in-cis* and *in-trans*.

### 3.1 Characteristics of *K. phaffii* lncRNAs

Our approach to lncRNA identification resulted in the identification of 168 lncRNAs, 13 of which were multi-exonic. In general, the identified lncRNAs were distributed across the genome, and apart from intronic lncRNAs, all classes were represented. Based on the reference annotation, only ∼70 genes could theoretically host an intronic lncRNA due to the lncRNA size cutoff of 200 nucleotides. The vast majority of lncRNAs annotated were antisense to other genes, with the majority of these lncRNAs fully containing their antisense gene.

The lncRNAs annotated in this study had a similar GC content and length to coding genes, metrics which are not widely reported from other yeast lncRNA annotations. In the *Candida* clade, the GC content and length of lncRNAs was lower than coding genes [50], while in *S. pombe* the GC content of a lncRNA has also been linked to the likelihood of the lncRNA function [49]. In mammalian cells, a significant correlation of GC content of lncRNAs with P-body accumulation has been reported [51] in addition to GC-rich sequences promoting nuclear export in a splicing independent manner [52]. The expression level of lncRNAs is significantly lower and distributed around that of other non-coding genes, which is supported by earlier reports in *K. phaffii* and in various other eukaryotic organisms [16], [23], [50].

As with other genome assemblies of *K. phaffii*, there are a number of unassembled gap regions in the CBS7435 genome [14], [53]. These are typically associated with highly repetitive regions, such as the sub-telomeric region. Many of those lncRNAs with several isoforms were located near sub-telomeric regions or gap regions, where repetitive sequences likely affect the mapping of reads.

### 3.2 Comparisons to previously reported lncRNAs

Two previous studies aiming to identify lncRNAs in *K. phaffii* have been reported [15], [16]. When comparing the lncRNAs annotated in these studies to our annotated lncRNAs, we found very little overlap, which may be due to their condition-specific expression, both studies looked at growth on methanol. Of the 10 differentially expressed lncRNAs (of the 56 found) reported by [15], we found 2 of them, including the annotation of a lncRNA (MSTRG.1706) nearby to the gene coding for pyruvate carboxylase (*PYC2*). Despite reporting > 200 lncRNAs, we could not retrieve all data of lncRNA annotations described by Sun and coworkers [16]. We looked at the genes neighbouring the 39 differentially expressed lncRNAs reported by Sun and coworkers [16], and found that we had annotated lncRNAs nearby to 21 of them. Similarly, of the 28 lncRNAs reported as antisense to a coding gene, we found 7 lncRNAs nearby to the reported coding gene but only one of these 7 lncRNAs was an antisense lncRNA. In addition to lncRNA expression being strongly condition dependent, the annotation of new transcripts is also dependent on sequencing technology and the reference annotation used, with the previous studies using an earlier GS115 annotation [9]. Considering that the vast majority of novel transcripts over 200 nucleotides long were predicted to be non-coding, the annotation of coding genes in the current CBS7435 annotation seems fairly comprehensive [14].

We also looked at lncRNAs reported in other yeasts. Earlier work in *S. cerevisiae* demonstrated that expression of an antisense lncRNA regulates expression of the phosphate transporter *PHO84* [54], [55]. Similarly, in the fission yeast *S. pombe,* expression of the phosphate transporter *PHO84* and acid phosphatase *PHO1* is inversely regulated by their respective lncRNAs, depending on phosphate availability [56]. In *K. phaffii*, a lncRNA (MSTRG.4225) was annotated upstream sense to the *PHO84–2* gene, but the genes were not correlated (r = 0.24, p = 0.389, n = 15). Additionally, a lncRNA (MSTRG.4426) was annotated nearby to *PHO88*, but was not correlated (r = -0.20, p = 0.475, n = 15). Neither the *PHO* genes nor the lncRNAs in their vicinity were differentially expressed in the retentostat conditions. In *S. pombe*, the expression of the *PHO* genes and their respective lncRNAs was only affected by varying phosphate conditions [56]. Thus, future work studying *K. phaffii* in alternative limiting conditions would likely reveal further potential lncRNAs and regulatory mechanisms. A lncRNA (MSTRG.3832) was annotated antisense to *PRP2*, which was also reported in *S. pombe*. *PRP2* encodes an RNA-dependent DExD/H-box ATPase required for the activation of the spliceosome. No differential expression of MSTRG.3832 was observed, but the expression of the *S. pombe* lncRNA increased during quiescence [57].

Two lncRNAs, *ICR1* and *PWR1,* have been described in *S. cerevisiae* as playing a crucial role in the regulation of *FLO11* [7]. *FLO11* is a member of the *FLO* gene family which are involved in cell-cell adhesion (flocculation) and filamentous growth. *FLO* gene expression is influenced by external factors like nutrients and stressors, contributing to various survival strategies like pseudohyphal growth, flocculation, and invasive growth. The mechanism of *ICR1* and *PWR1* enables a toggle-like switch of *FLO11* expression. We looked at the assembled transcripts near *FLO11* of *K. phaffii* and found no evidence for a similar mechanism in the upstream region of *FLO11*. However, a transcript (that was annotated as coding by RNAsamba and thus discarded) was annotated on the antisense strand of *FLO11,* which converges with the *FLO11* transcript and overlaps it at its 3’ end (MSTRG.1995). Neither *FLO11* nor the transcript (MSTRG.1995) were differentially expressed in the comparison between 0.025 and R10. Whether the transcript MSTRG.1995 is non-coding or not, or whether it is involved in regulating *FLO11* expression, remains to be determined in future. Although the strain used in the retentostat experiment was deficient in Flo8, the main transcriptional regulator of *FLO* genes, which may affect our extrapolation of the results to a wild-type strain, we would still expect the *ICR1* and *PWR1* lncRNAs to be detected if present. We did however annotate several other lncRNAs in the vicinity of *FLO* family genes, including *FLO200*, *FLO5–2*, *FLO100*, and PP7435_Chr3–1237, all of which are sub-telomeric [14], [58]. Considering the annotation of at least 4 lncRNAs near the *FLO* genes in *K. phaffii*, we suspect that also in *K. phaffii*, lncRNAs play a wider role in the transition to pseudohyphal growth. Despite none of the 4 lncRNAs being differentially expressed in the analysed conditions, their annotation warrants future investigation to better understand this complex, biotechnologically relevant phenotype in *K. phaffii*.

The most strongly upregulated lncRNA at slow growth rates was MSTRG.3443. It is antisense to the gene *AGP2–2,* which was strongly downregulated (Supplementary Figure 2). Agp2–2 is structurally similar to transporters but lacks transport activity. Instead, it acts as a sensor and transduces environmental signals via its regulatory effects on the transcription of many transporter genes. In *S. cerevisiae*, a reduction in expression levels of various transporters, including the low affinity hexose transporter *HXT3,* has been reported in a *Δagp2–2* strain [59]. For several transcripts regulated by Set3 histone deacetylase in *S. cerevisiae* (including *AGP2* and *GAL1*), it was demonstrated that high antisense transcription disrupts Set3 deacetylase activity, leading to altered chromatin architecture and reduced sense transcript production in a chromatin-dependent manner [60], [61]. Based on these reports, we speculate that transcription of MSTRG.3443 may lead to H3K4me2 accumulation in the *AGP2–2* promoter region, leading to deacetylation of the promoter by Set3 and subsequent downregulation of *AGP2–2*, potentially leading to the downregulation of many transporter genes. Indeed, more than half of the 179 genes annotated as transporters in the *K. phaffii* genome were differentially expressed in the retentostat in at least one sampling point, with 39 of them having lower expression at slower growth rates [22]. These shifts in transporter activity may reflect broader metabolic rewiring at near-zero growth, suggesting a potential—though speculative—role for MSTRG.3443, and other lncRNAs, in mediating energy conservation during adaptation to substrate-limited conditions [21].

### 3.3 Potential functions and roles of lncRNAs at near-zero growth

lncRNAs are not widely conserved between species, with reports from *S. cerevisiae* demonstrating strain-specific responses [62]. However, despite this lack of conservation, the mechanisms and involvement of lncRNAs in regulatory functions may be conserved. In this regard, regulatory interactions of lncRNAs annotated near previously reported lncRNA-associated genes are interesting. Similar to the approach used by Wolf and coworkers [62], we connected the lncRNAs and other coding genes to study their potential *cis* and *trans* interactions, finding lncRNA expression levels were more positively correlated to the expression level of genes in their vicinity than with independent genes. This may be explained by a regulatory mechanism; but could equally be attributed to a more open chromatin region simultaneously affecting the expression of a lncRNA and nearby genes. In contrast, negative correlation is more likely to suggest an involvement of the lncRNA in the downregulation of the nearby gene. *Trans* interacting pairs were identified based on triplex formation propensity. The observation of a bimodal density distribution of the correlation coefficients between lncRNAs and predicted interaction partners, suggests the involvement of lncRNAs in robust regulatory mechanisms.

There is likely some species-specific regulatory mechanisms in *K. phaffii*, particularly given it’s special ecological niche. Interestingly, we identify some lncRNAs in vicinity to genes involved in methanol utilisation such as MSTR.5282 (divergent to *AOX2,* both being strongly upregulated), MSTRG.2087 (antisense of *TAL1–2* encoding peroxisomal transaldolase) and MSTRG.3459 (downstream antisense of dihydroxyacetone synthase isoform *DAS2*). While the latter two lncRNAs are not differentially expressed in the analysed glucose-limited conditions, their presence indicates a potential regulatory feature during growth on methanol. Furthermore, many of the differentially regulated lncRNAs are antisense to genes with so far uncharacterised functions.

MSTRG.2225 is divergent to *YHP1* and is among the six lncRNAs downregulated at decreasing growth rates. In *S. cerevisiae*, *YHP1* encodes a homeoprotein that represses early cell cycle box containing M/G1 target genes [63]. It also represses *IME1* expression, the master regulator of meiosis [64], whose transcription is also inhibited in *cis* by transcription of an lncRNA in it's promoter [65]. *IME1* is not present in *K. phaffii* and other non-conventional yeasts such as *Ogataea polymorpha* or *Candida krusei* [64], [66], [67]. In addition to the downregulation of MSTRG.2225, *YHP1* was also strongly downregulated at slow growth rates, while *IME2*, which in *S. cerevisiae* is activated by Ime1, was upregulated. A lncRNA (MSTRG.46) was annotated upstream sense (496 nt) to *IME2* but was not differently expressed. MSTRG.3382 was strongly downregulated at later timepoints in the retentostat and is annotated as divergent to *SET5*, which cooperates with Set1 to repress gene expression at telomeres and retrotransposons [68]. lncRNAs were also annotated antisense to many other meiosis related genes, including *SPO11* (MSTR.1041, upregulated), *SPO71* (MSTR.1325) and *RDH54* (MSTR.1633). The lncRNA MSTR.2774 was also annotated as antisense to *MFA2* encoding the mating pheromone factor-a. Thus, there is strong indications for the involvement of lncRNAs in mitosis and meiosis in *K. phaffii*, however, their mechanisms remain unclear and except for MSTR.1041 none were differentially regulated in the analysed conditions.

The most strongly downregulated antisense lncRNA, MSTRG.1680, is antisense to *LIH1–2* (PP7435_Chr1–1564) and divergent to PP7435_Chr1–1565 (Supplementary Figure 3). LIH1–2 encodes a lipase that converts tri-, di- and monoglycerides of long-chain fatty acids into fatty acids and glycerol [69]. PP7435_Chr1–1565 is a hypothetical protein that contains Armadillo-like repeats and provides a weak hit to a bud-emergence protein. A possible mechanism may involve the activation of both MSTRG.1680 and PP7435_Chr1–1565, with MSTRG.1680 subsequently repressing *LIH1–2*; allowing for coordinated control of lipid metabolism and growth-dependent cellular processes. Furthermore, processes related to lipid or fatty acid metabolism were enriched in the interacting partners of three of the lncRNAs, albeit not for MSTRG.1680. These findings suggest an involvement of lncRNAs in our previously observed shift in lipid metabolism toward storage and membrane adaptation under extremely slow growth conditions [22].

Overall, our findings show that *K. phaffii* contains a diverse collection of lncRNAs, the expression of which is closely tied to growth rate dependent regulation. Their varied modes of action highlight their potentially important roles in the adaptation of *K. phaffii* to challenging conditions such as near-zero growth. These insights expand our understanding of lncRNA-mediated regulation in non-conventional yeasts and enable the potential leveraging of lncRNAs in the optimisation of industrial bioprocesses.

## 4 Materials & Methods

The code used for analysis and necessary data for reproduction are available on GitHub at https://github.com/bcoltman/Kphaffii_lncRNA_NearZero. A snapshot of this repository can be found on Zenodo at: https://doi.org/10.5281/zenodo.15148334. A separate upload to Zenodo including the files and results generated by the scripts and presented in this manuscript can be found here: https://doi.org/10.5281/zenodo.15148207. Unless specified, the default parameters for each programme were used.

### 4.1 RNA-Seq pre-processing

The RNA-seq data used in this study were obtained from a previously published investigation exploring recombinant protein secretion in *Komagataella phaffii* CBS2612 under glucose-limited conditions across a wide range of growth rates [22]. For detailed experimental procedures and growth physiology, readers are referred to the original publication [22]. In summary, a total of 24 RNA-seq libraries were generated from a vHH-secreting *K. phaffii* strain cultivated at growth rates spanning over 200-fold (0.00047–0.1 h⁻¹). Libraries were sequenced using paired-end 150 bp reads at a depth exceeding 24 million read pairs per sample. Sampling was performed across eight time points: one from a steady-state chemostat at a high growth rate (0.1 h⁻¹, C0.1, 3 replicates), and seven from retentostat cultivations initiated from chemostats operated at 0.025 h⁻¹. Retentostats were initiated after either two weeks (short chemostat, SC, 3 replicates) or three weeks (long chemostat, LC, 3 replicates) of chemostat cultivation. Samples were collected at the start of the retentostat phase (0.025), and approximately ∼6 (R3), ∼14 (R6, SC only), and ∼28 days (R10) post-initiation. In the SC cultivations, regression models of the retentostat cultivation predicted growth rates of ∼0.0022 h^−1^, ∼0.0010 h^−1^ and ∼0.0005 h^−1^ at sampling points R3, R6 and R10, respectively [21], [22].

Raw RNA-seq reads were downloaded from the NCBI Sequence Read Archive (SRA accession: PRJNA1013119). Low quality bases and contaminating adapter sequences were removed using cutadapt v3.2 [70] and Trimmomatic v0.36 (SLIDINGWINDOW of 4 with quality > 20, MINLEN of 36) [71].

### 4.2 CBS7435 reference annotation updates

Building on the most recent published annotation of CBS7435 [14], the gene annotation of CBS7435 was further refined using insights from a novel de novo gene prediction of CBS2612 (unpublished). This prediction was generated through a collaborative effort employing the gene prediction tool Augustus [72], [73] alongside RNA-Seq data (GEO Series accession GSE111973) [74]. Manual curation complemented this approach, enabling the integration of high-confidence gene models from the type strain CBS2612 as a reference, significantly enhancing the accuracy and completeness of the CBS7435 annotation. The CBS7435 reference genome consists of the genome sequence downloaded from NCBI (GCA_000223565) and this updated annotation.

### 4.3 RNA-Seq mapping and transcriptome assembly

The CBS7435 reference genome was indexed using STAR v2.7.11b (sjdbOverhang = 149, genomeChrBinNbits = 16, genomeSAindexNbases =10) before the reads were mapped with parameters as defined by the ENCODE project [75] (--outFilterMultimapNmax 20, --alignSJoverhangMin 8, --alignSJDBoverhangMin 1, --outFilterMismatchNmax 999, --outFilterMismatchNoverReadLmax 0.04, --alignIntronMax 1000000, --alignMatesGapMax 1000000, --alignIntronMin 20, --outFilterType BySJout, --sjdbScore 1) [24].

StringTie v2.2.1 was used for genome guided transcriptome assembly of the aligned RNAseq reads, requiring 5 reads to support a junction and 3 % junction read support [25]. The resulting annotations were merged into a non-redundant set with a minimum isoform fraction of 10 % and a minimum transcript coverage of 10.

To filter out already annotated genes, transcripts that overlapped with an exon (same-strand) of a gene (coding or non-coding) were removed using the intersect function from BEDtools v2.28.0 [76]. Transcript per million (TPM) expression values for the lncRNA transcripts were calculated using the Salmon quantmerge function Salmon v1.10.0 [36].

### 4.4 lncRNA annotation

An overview of the transcript filtering and classification workflow is shown in Fig. 1, with each step described in detail below. Transcripts < 200 bp were discarded prior to coding potential analysis. Remaining transcripts were initially assessed using the default mode (randomly shuffled mRNAs) of FEELnc v0.2.0 to distinguish coding from non-coding [26]. FEELnc was also used for classification of lncRNAs with respect to protein-coding genes.

To further evaluate coding potential, several tools were compared: CPC2 v1.0.1 [29], RNAsamba v0.2.5 [28] and CPAT v3.0.2 [30]. These tools were benchmarked using the CBS7435 reference annotation to evaluate classification performance. For each tool, we computed the following values: true positive (TP) represents a correctly identified coding sequence, true negative (TN) a correctly identified non-coding sequence, false positive (FP) as a non-coding sequence predicted as coding and a false negative (FN) as a coding sequence predicted as non-coding. Performance was assessed using: Balanced Accuracy = $\frac{\text{Recall }+\text{ Specificity}}{2}$, where Recall $=\frac{\text{TP}}{\text{TP}+\text{FN}}$ and Specificity $=\frac{\text{TN}}{\text{TN}+\text{FP}}$, and F1 score $=\;2\times \frac{\text{Precision}\times \text{Recall}}{\text{Precision}+\text{Recall}}$ where Precision . Here the training sets for RNAsamba included: the FEELnc shuffled non-coding regions from the CBS7435 annotation; additional non-coding/coding transcripts from the CBS7435 annotation; and the balanced, integrated coding and non-coding sequences released by CPPred [27].

Based on performance, we selected RNAsamba for the final classification of the transcripts. An ensemble model of two RNAsamba models were applied: the pre-trained RNAsamba model and a model trained using the CPPred dataset extended with CBS7435 sequences. A transcript was retained if classified as non-coding by both FEELnc and the RNAsamba ensemble model. CPAT and CPC2 results were assessed but not used to exclude transcripts.

To identify possible functional ORFs, transcripts were analysed using TransDecoder v5.5.0 [31], retaining translated open reading frames (ORFs) > 100 amino acids. Resulting protein sequences were queried against SWISSPROT [77] (release version 2024_06) using BLASTP, and domain content assessed with hmmscan [32] against the PfamA v37.1 database [33]. Hits with an E-value < 1e-5 were considered significant.

Nucleotide sequences of all putative lncRNAs were queried using RNAcentral, including integrated searches of RFAM [34], [35]. Hits to RFAM database with E-value < 1e-5 were used to identify known structured RNAs, which were excluded. High similarity RNACentral hits (identity > 90 %, E-value < 1e-5) were excluded unless manual inspection identified discrepancies with current genome annotations (e.g. MSTRG.1715, which was retained due to reannotation context; see Results). Less stringent RNACentral hits (>50 % identity, E-value < 1e-2) were used to compare reported RNA types and their origin but were not used for filtering.

Finally, expression filtering was performed using the transcript-level quantifications calculated using the quantmerge function from Salmon v1.10.0 [36]. Transcripts with < 1 TPM in all samples were excluded.

The intersection of all filtering steps – length threshold, coding potential, domain, and RFAM filtering, and expression threshold – yielded the final set of 190 lncRNA transcripts, from 168 loci (Supplementary File 1).

### 4.5 Read counts

A reference transcriptome was extracted from the Stringtie merged transcriptome using gffread v0.12.6 [78]. For the retentostat cultivations, this included the antibiotic resistance and recombinant vHH transcripts [22]. These transcriptomes were indexed using Salmon v1.10.0 [36] and also the complete chromosomal sequences were included as a decoy. Reads were pseudo aligned and quantified using Salmon with 100 bootstrap samples and including the –gcBias flag (accounting for sample specific biases e.g. fragment-level GC bias) and –seqBias flag (accounting for sequence-specific effects possible accumulated during sample prep). Samples from the LC cultivation type were only used for annotating and filtering (TPM) of lncRNAs. The samples were excluded from all later analysis.

Results were imported into R v4.3.2 and processed using IsoformSwitchAnalyzeR (ISAR) v2.2.0 [79]. ISAR was used as it provides a wrapper around tximport [80] to process the Salmon estimated counts and abundances, but also addresses some Stringtie annotation specific issues with merging of nearby/overlapping transcripts. Gene counts were merged with ISAR from the transcript expression counts, rounded to the nearest integer and imported into DESeq2 v1.42.0 [81].

### 4.6 Differential gene expression

Differential gene expression was analysed using DESeq2 [81] with two complementary approaches: a time-course analysis using a likelihood ratio test (LRT) to identify genes generally associated with growth rate, and pairwise Wald tests for specific comparisons between sampling points (i.e. *growth rates*).

Growth rate association was assessed using the Likelihood Ratio Test (LRT) by comparing two models for each gene: a full model that includes the effect of sampling point (∼ sampling point), representing different growth rates, and a reduced model without this factor (∼ 1). The LRT determines whether the inclusion of growth rate significantly improves the explanation of expression variability. Genes were considered as associated with growth rate if the Benjamini-Hochberg adjusted p-value (padj) was less than 0.0005.

Specific growth rate dependent expression changes were identified using pairwise Wald tests. Pairwise comparisons were limited to samples within the same SC cultivation type. The C0.1 chemostat was not included in the analysis to avoid potential batch effects that could not be controlled for. Thus the model accounted for the sampling points (i.e. *growth rates*) and all pairwise comparisons were made relative to the 0.025 sampling point. Wald tests were performed using shrunken log2 fold-change estimates obtained with apeglm [82] and a log-fold change threshold of 0 was applied. Genes were considered differentially expressed if the s-value was less than 0.005.

### 4.7 Correlation and clustering of counts

A design blind, variance stabilising transformation (VST) was performed on the gene counts matrix using DESeq2. Rows (genes) with uniform values were removed, resulting in a post-VST matrix. These uniform values are an artefact from the process of estimating abundances with Salmon. The correlation matrix of all annotated genes across all samples was calculated from this post-VST matrix by z-score standardisation of the rows, before calculating the Pearson correlation coefficient using the “cor” function.

Genes were clustered according to both expression level and profile, using MPLNclust; or according to expression profile alone, using Mfuzz. In both cases, genes that were not identified as growth rate associated in a likelihood ratio test (LRT, see Differential Expression above) were not included in clustering.

For clustering according to the expression profile, gene expression profiles were soft clustered by Mfuzz [37]. The post-VST matrix was reduced to only include genes that were growth rate associated (LRT) and the median of the replicates at the sampling points was determined. The expression levels of each gene were then standardised with Mfuzz (standardize). Between 2 and 20 clusters were assessed; none of the resulting clusters were empty, but an optimal model of 4 clusters was selected based on the results of the Dmin function, which was repeated 10 times and returns the average minimum centroid distance between two cluster centres. Four clusters were selected based on the observation that adding more clusters resulted in only a minimal decrease in the average distance between centroids. A cutoff of 0.7 was used for determining cluster membership.

For clustering according to expression level and profile with MPLNclust, the median of the raw counts for each gene was determined. This median-counts matrix was used directly by MPLNclust, with the mixture models fitted via variational inference [38], [39]. Initial clustering was performed via kmeans, and the best-fitting model up to 20 clusters was assessed. The optimal model was determined by consideration of the log-likelihood, the Bayesian information criterion (BIC), Akaike information criterion (AIC), a variation of AIC (AIC3) the integrated completed likelihood (ICL) and Data-Driven Slope Estimation (DDSE). A model of 7 clusters was selected based on consideration of these optimality criteria. Cluster membership for MPLNClust clustering was based on the most likely cluster label.

### 4.8 Enrichment analysis

Enrichment analysis was performed using g:Profiler, using custom Gene Matrix Transposed (GMT) files [83]. Two analyses were performed, assessing enrichment of gene ontology (GO) and KEGG pathways. A GO GMT file for the *K. phaffii* GS115 annotation, based on the annotation available in Ensembl was downloaded from g:Profiler. KEGG pathway analysis is normally available in g:Profiler; however, the KEGG pathway annotations are not included in the downloadable files. Therefore, the KEGG pathway file for the GS115 annotation was downloaded from KEGG [84]. In both files, the GS115 identifiers were converted to their CBS7435 identifiers based on the annotated homologs [14]. These files were then used as custom GMT files for their respective analysis.

### 4.9 Triplex forming lncRNA-gene pairs

Triplex target sites (TTSs) within the CBS7435 genome sequence and corresponding triplex forming oligos (TFOs) within the lncRNA sequences were predicted with Triplexator v1.3.2 [42]. Default settings were used, apart from: a minimum triplex length of 20, a maximum error rate of 20 %, 2 consecutive errors permitted, a minimum guanine content of 20 %, a maximum error rate of 5 %, and merging of overlapping sites. To ensure that each triplex target site (TTS) was assigned to a unique genomic feature, we implemented an exclusive, priority-based classification scheme using intersect from BEDtools v2.28.0 [76]. The genome was classified in a strand-independent manner into either promoter, exon, intron or intergenic regions based on the intersection with the respective features. The region 1000 bp upstream-sense or 250 bp downstream-sense of a transcription start site was defined as a promoter region; followed by exons, then introns or if no features intersected the region then it was defined as intergenic. The intersection of TTSs with these regions were then assessed. If a TTS overlapped two types of region, then priority was given in the same order: promoters, exons, introns or intergenic. This sequential approach ensured that each TTS receives a single classification. Unique, interacting lncRNA-coding genes pairs were identified from the results.

### 4.10 Neighbouring lncRNAs-genes

To find genes within 2000 bp of the lncRNAs, the window function of BEDtools v2.28.0 was used [76]. The resulting bed file was parsed to extract unique neighbouring lncRNA-gene pairs. Pair-wise (interacting pairs e.g. via triplexes, or neighbouring) heatmaps of standardised expression values were plotted using ggplot2 [85] and ComplexHeatmaps [86].

### 4.11 Coverage plots

Coverage plots of representative lncRNAs were plotted with karyoploteR [87]. Aligned reads from STAR were separated to strand specific bam files and used for plotting with the kpPlotBAMCoverage function. The highest coverage peak of the plotted regions was determined first with the unseparated BAM file, before plotting the strand-specific coverage values.

## CRediT authorship contribution statement

**Coltman Benjamin Luke:** Writing – review & editing, Writing – original draft, Methodology, Investigation, Formal analysis, Data curation, Conceptualization. **Motheramgari Krishna:** Writing – review & editing, Methodology, Formal analysis. **Tatto Nadine:** Writing – review & editing, Data curation. **Gasser Brigitte:** Writing – review & editing, Supervision, Funding acquisition, Formal analysis, Conceptualization.

## Declaration of Competing Interest

The authors declare that there are no competing interests.

## Footnotes

Appendix A Supplementary data associated with this article can be found in the online version at doi:10.1016/j.csbj.2025.04.028.
