# Supplementary material for: Identification and functional analysis of growth rate associated long non-coding RNAs in Komagataella phaffii
Source: Comput Struct Biotechnol J. 2025 Apr 22;27:1693–705. doi: 10.1016/j.csbj.2025.04.028 (PMC12063151; doi:10.1016/j.csbj.2025.04.028)
Supplement: Supplementary file 1 — Supplementary material [file mmc1.docx]

# Supplementary Information

Supplementary Table 1: Comparison of different coding potential prediction tools to discern coding and non-coding transcripts annotated in the CBS7435 genome. Values in the first two columns represent incorrect predictions. RNAsamba was trained with three different datasets: the shuffled mRNA data generated by FEELnc, the balanced CPPred integrated dataset and the pre-trained model distributed with RNAsamba. This pre-trained dataset is optimal for transcripts annotated from de-novo annotations and was trained on both full and partial length transcripts. CPAT was trained using the CPPred integrated dataset, whilst CPC2 is pre-trained and was not updated.

| Data set | Coding, out of 5425 | Non-coding, out of 250 | Balanced Accuracy | F1 score |
| --- | --- | --- | --- | --- |
| RNASamba - pre-trained, partial-length | 89 | 25 | 0.9418 | **0.9894** |
| RNASamba - FEELnc shuffled | 463 | 6 | 0.9453 | 0.9549 |
| RNASamba - CPPred integrated | **126** | **1** | **0.9864** | 0.9882 |
| RNASamba - Ensemble (all of above 3) | 148 | 1 | 0.9844 | 0.9861 |
| CPAT (using CPPred integrated) | 290 | 2 | 0.9693 | 0.9724 |
| CPC2 | 333 | 1 | 0.9673 | 0.9682 |


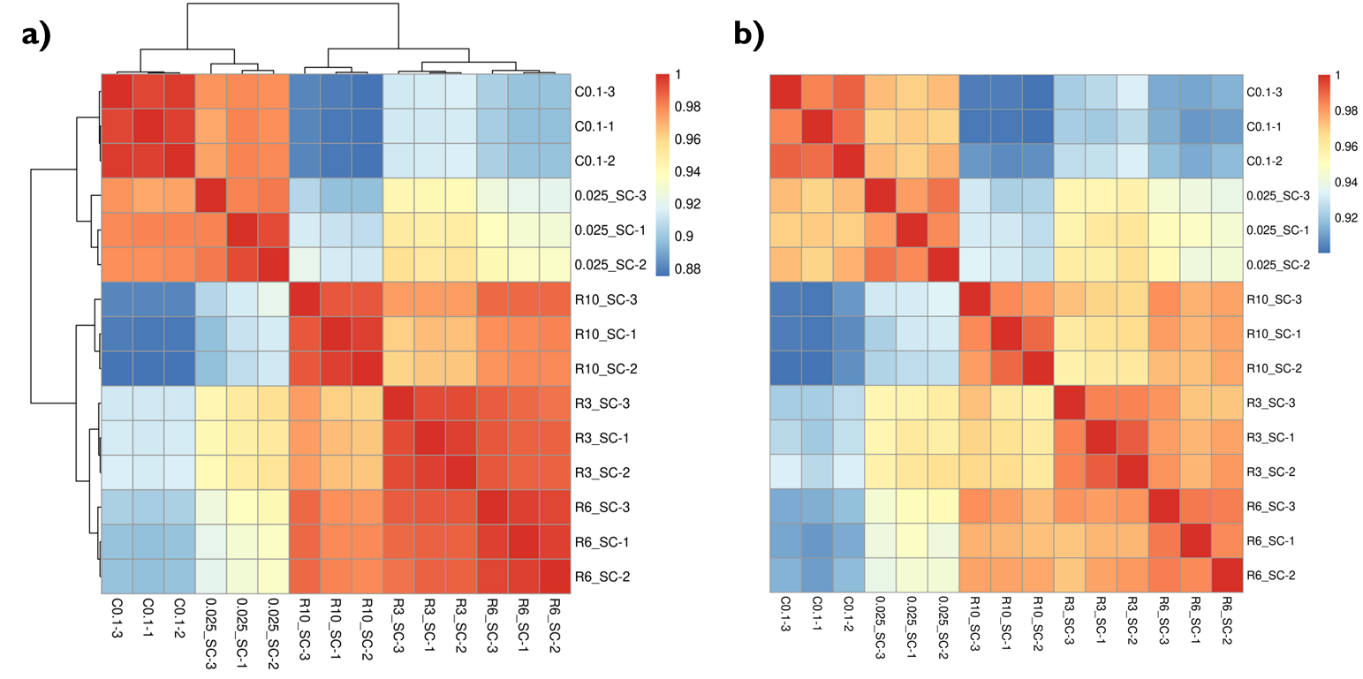


Supplementary Figure 1: Hierarchical clustering of samples based on a) all gene counts and b) only lncRNA counts.


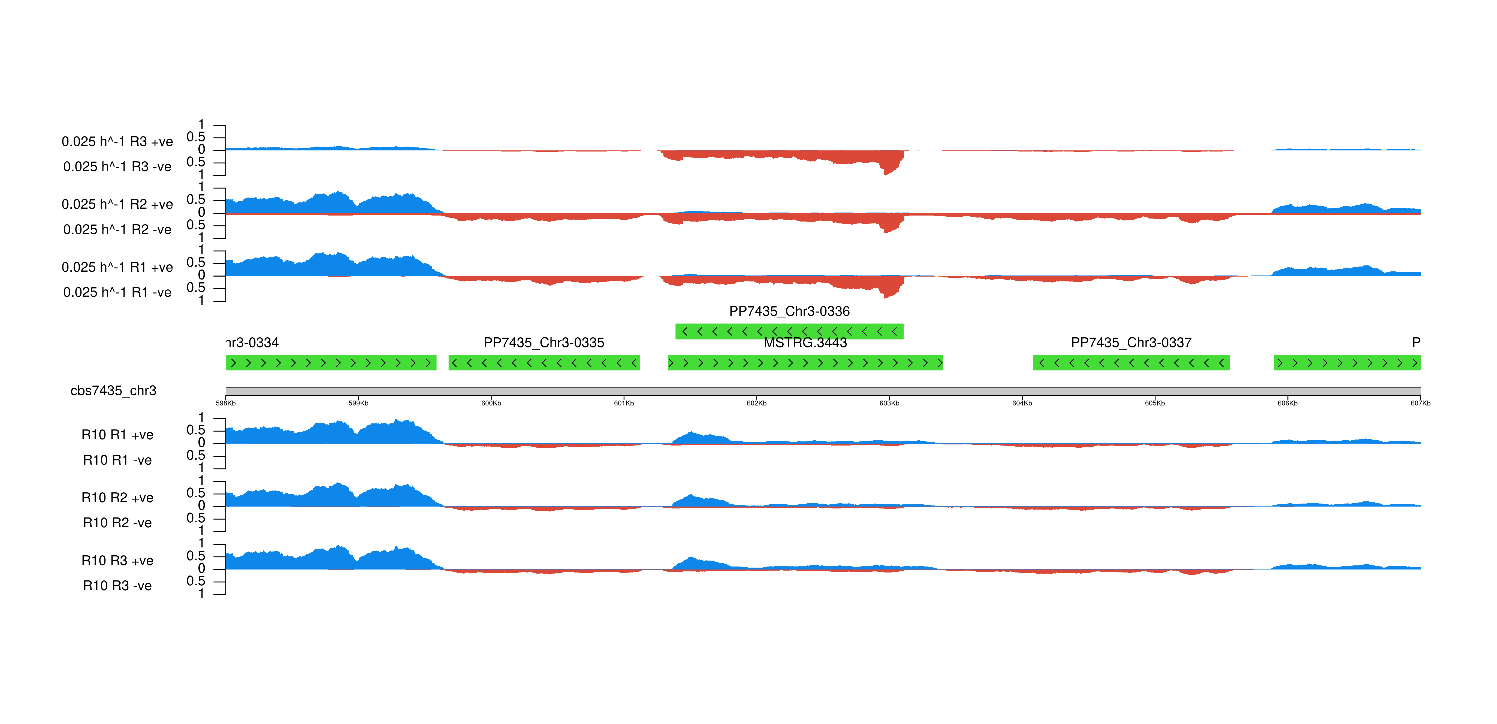


Supplementary Figure 2: Growth rate dependent changes of the region containing MSTRG.3443. Coverage plot of reads mapped to the positive (blue) and negative (red) strand for the three replicates at sampling point 0.025 (0.025 h^-1^, top three subplots) and R10 sampling point (0.0005 h^-1^, bottom three subplots). Within each subplot, the positive and negative strand are scaled to the highest coverage peak across both strands and within the plotted region. The Y-axis in each subplot is also normalised to this highest coverage peak. The central plot presents the architecture of the genome region. Transcripts originating from the positive and negative strand are annotated with forward (>) or reverse (<) arrows, respectively. PP7435_Chr3-0034=BBC1; PP7435_Chr3-0035=SSK1; PP7435_Chr3-0336=AGP2-2; PP7435_Chr3-0337=Protein with similarity to YKL162C.


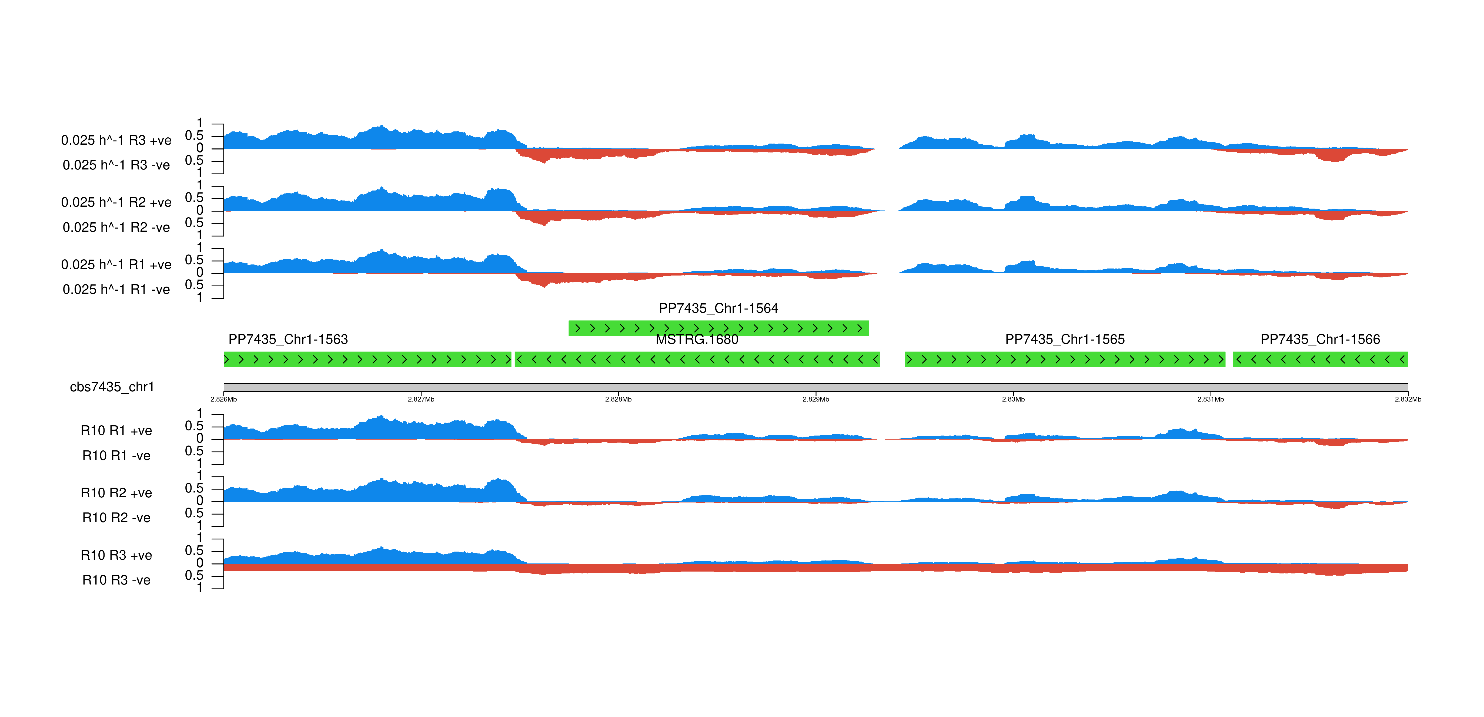


Supplementary Figure 3: Growth rate dependent changes of the region containing MSTRG.1680. Coverage plot of reads mapped to the positive (blue) and negative (red) strand for the three replicates at sampling point 0.025 (0.025 h^-1^, top three subplots) and R10 (0.0005 h^-1^, bottom three subplots). Within each subplot, the positive and negative strand are scaled to the highest coverage peak across both strands and within the plotted region. The Y-axis in each subplot is also normalised to this highest coverage peak. The central plot presents the architecture of the genome region. Transcripts originating from the positive and negative strand are annotated with forward (>) or reverse (<) arrows, respectively. PP7435_Chr1-1563=SKY1; PP7435_Chr1-1564=LIH1-2; PP7435_Chr1-1565=Hypothetical protein containing Armadillo-like repeats; PP7435_Chr1‑1566=YPC1.


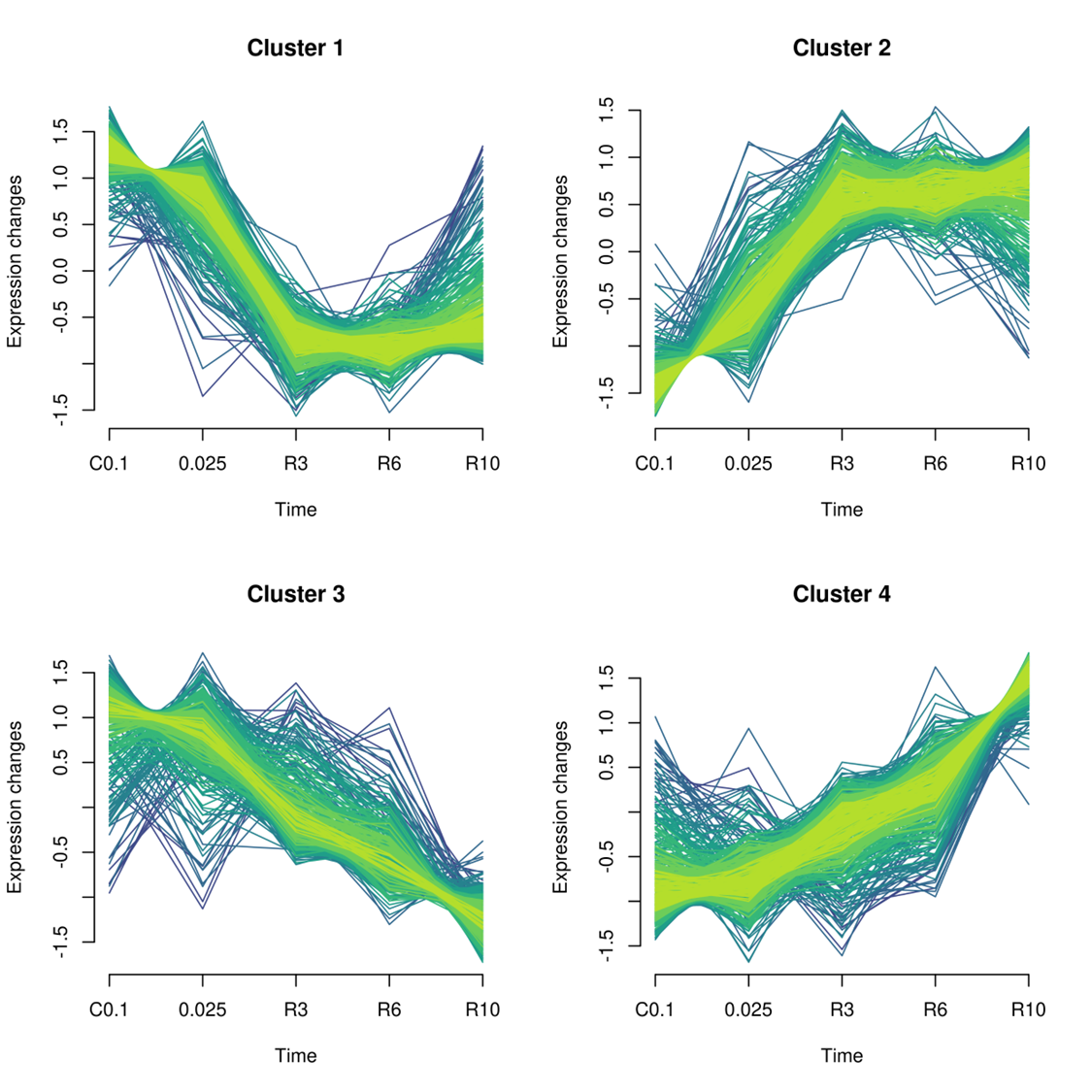


Supplementary Figure 4: Expression profiles of the different clusters. The z-score normalised values of each gene are connected by lines, which are coloured according to the membership value of the gene and cluster. As Mfuzz uses a soft clustering approach, a gene can belong to multiple clusters, with the membership value ranging between 0 and 1 and summing to 1 across all sampling points. Only genes with a membership value above 0.7 for a cluster are plotted, with yellow demonstrating a high membership value and blue a lower value.


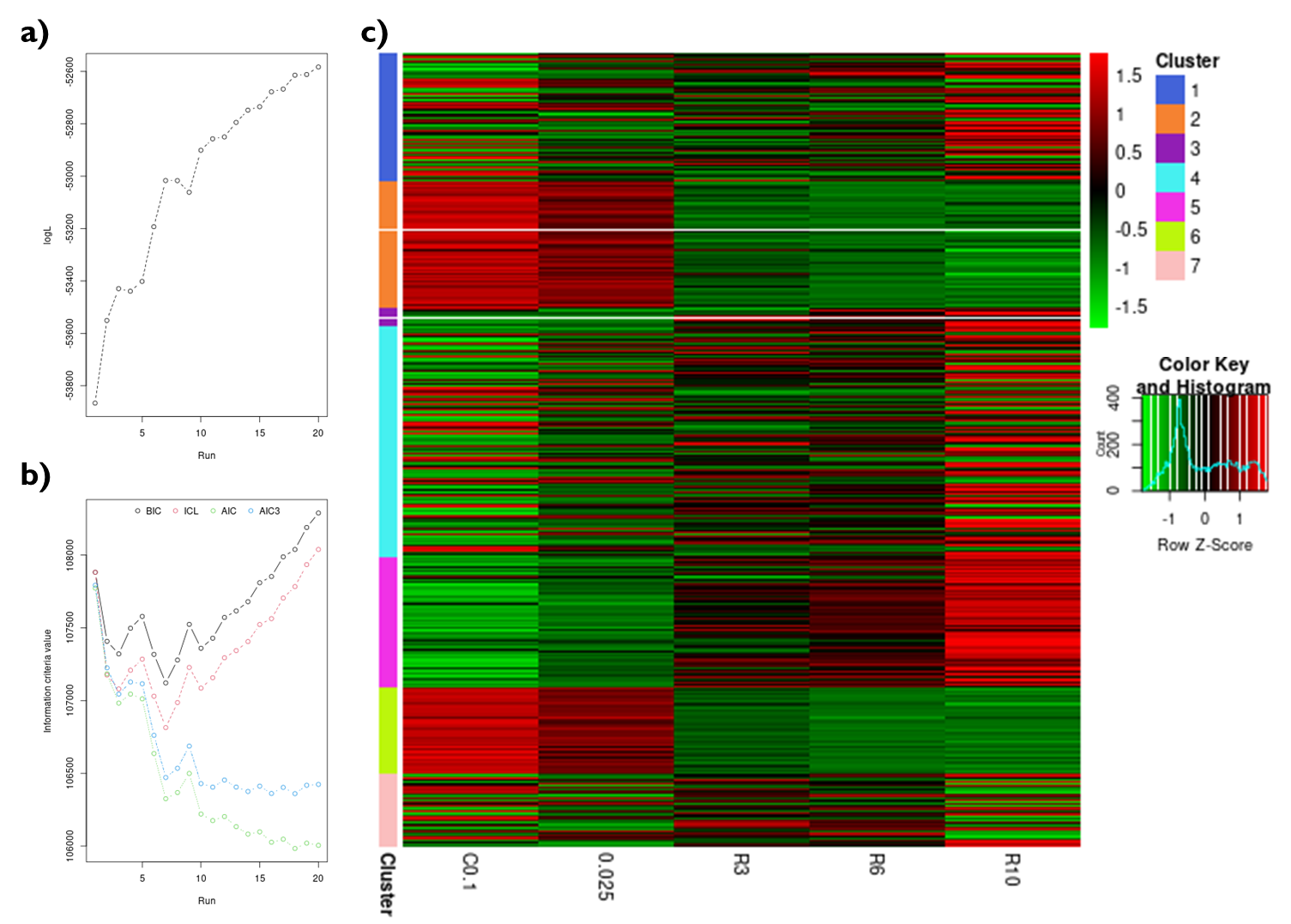


Supplementary Figure 5: Clustering of gene counts by a multivariate Poisson log-normal mixture model using MPLNClust. a). Selecting the number of clusters according to the a) log-likelihood of the different models and b) different information criteria. c) Heatmaps of z-score normalised expression levels, with genes along rows and samples along the columns, where the group membership is shown on the left-hand side. Only genes that demonstrated a growth rate dependent differential expression, as determined by a likelihood ratio test, were used in clustering. The optimal number of clusters are balance between model complexity and the log-likelihood of the fitted model.


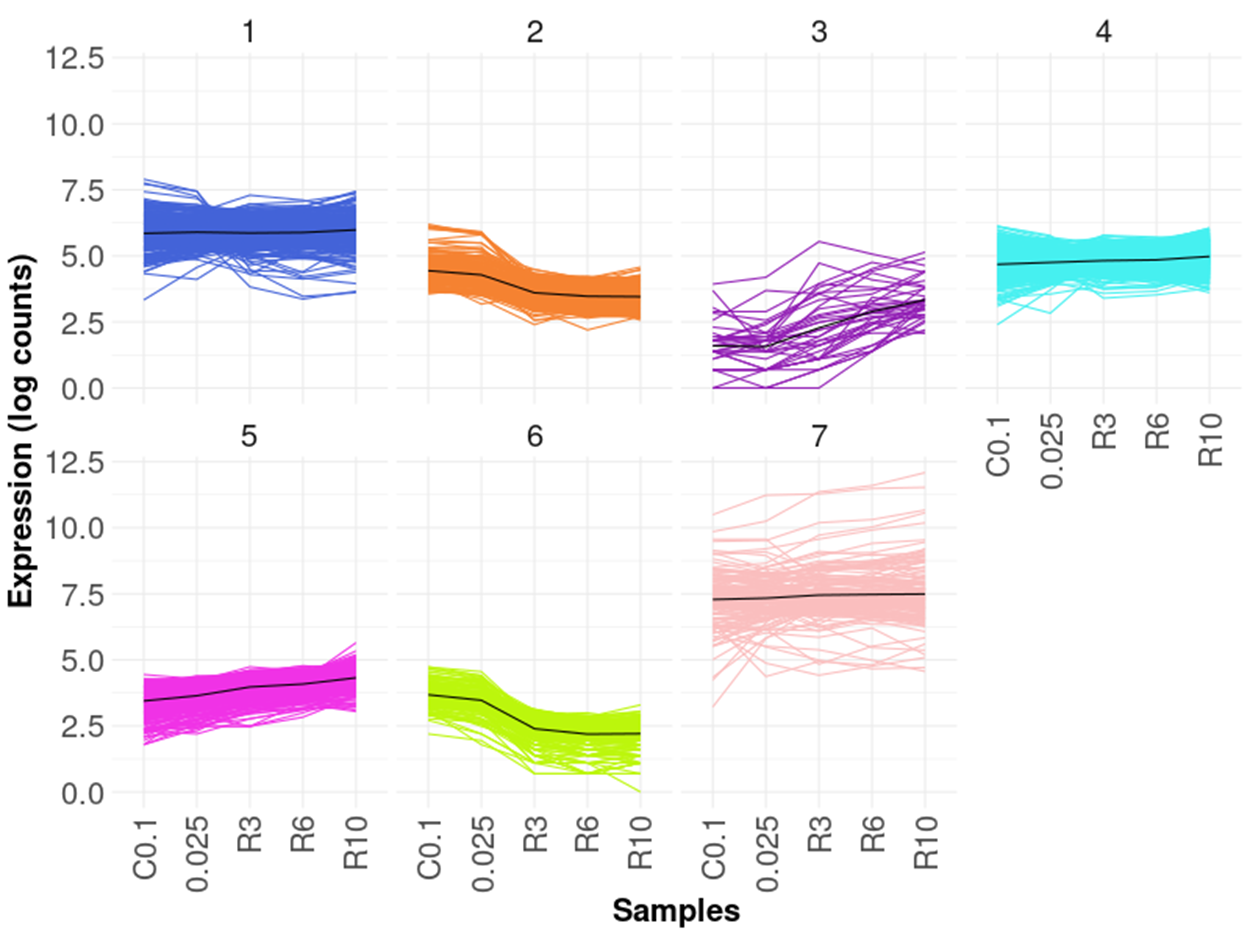


Supplementary Figure 6: Expression patterns of the 7 clusters identified via MPLNClust. The expression represents the log-transformed counts. The black lines represent the mean expression level for each cluster. Colouring of the clusters as in Supplementary Figure 5.


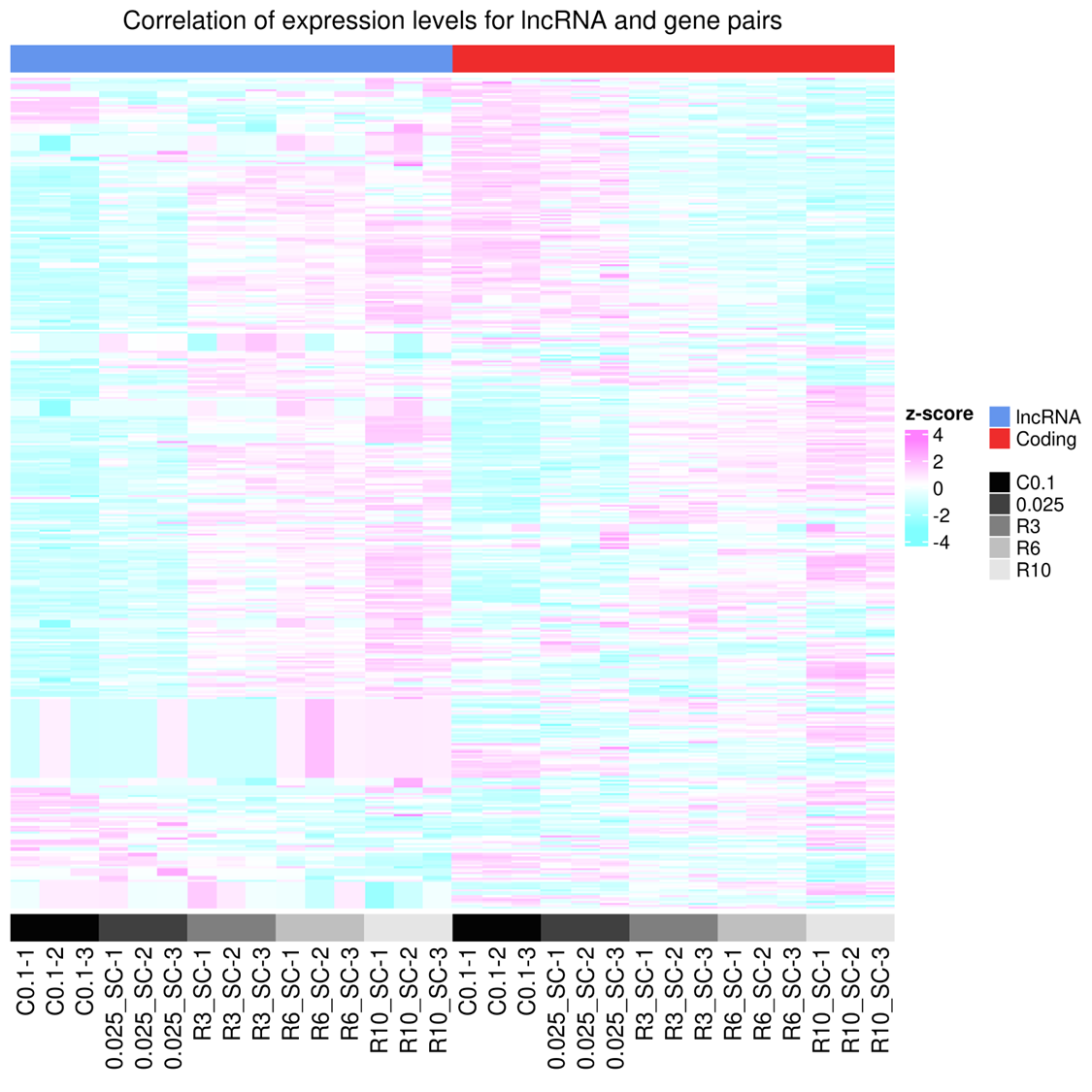


Supplementary Figure 7: Heatmap of expression profiles of lncRNA and interacting (via TTS in the promoter region) gene pairs. Each column represents a sample, with the left set of the columns representing the lncRNA, and the right set of columns the genes. Each row represents a lncRNA-gene pair, with the z-score normalised expression levels of the lncRNA or gene plotted.


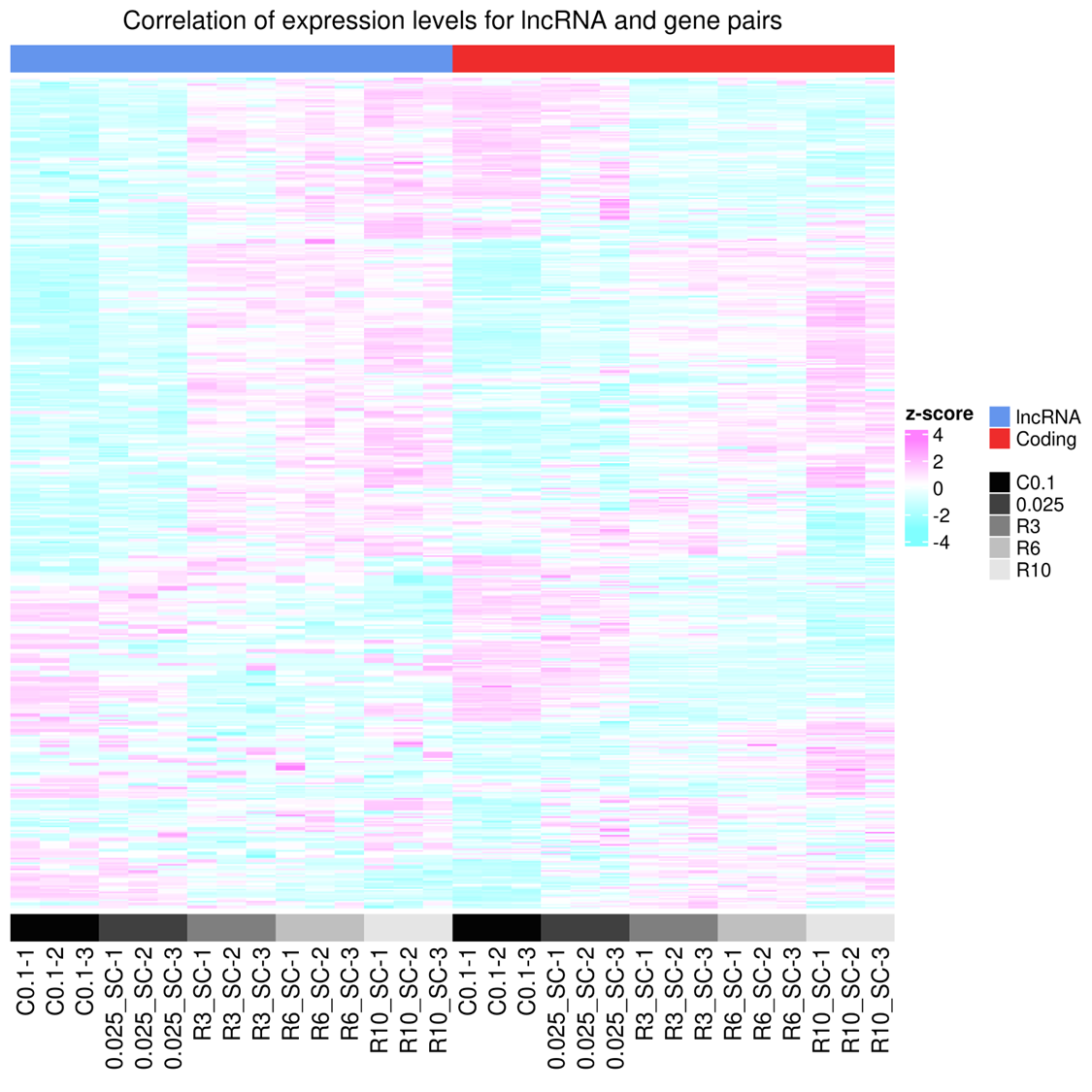


Supplementary Figure 8: Heatmap of expression profiles of lncRNA and neighbouring (2 kb) gene pairs. Each column represents a sample, with the left half of the columns representing the lncRNA, and the right set of columns the genes. Each row represents a lncRNA-gene pair, with the z-score normalised expression levels of the lncRNA or gene plotted.


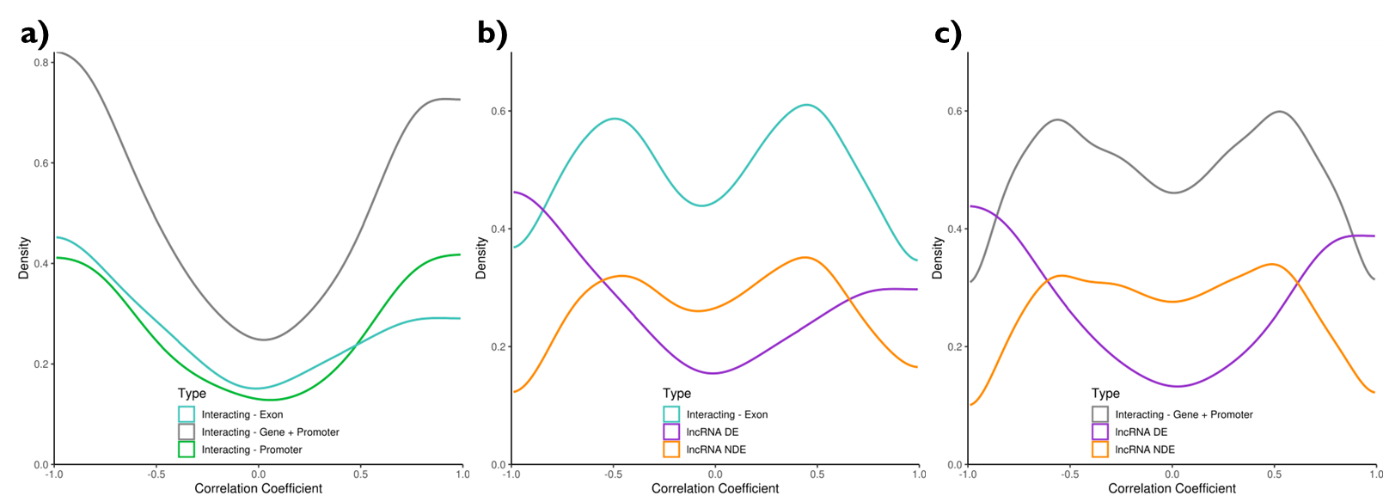


Supplementary Figure 9: Correlation coefficients of lncRNA and coding genes. Density plots of the correlation coefficient of: a) coding genes and growth rate associated lncRNAs interacting via triplexes. Different coloured lines represent whether the distribution includes the correlation coefficient of lncRNAs and coding genes predicted to interact with each other via triplexes in the promoter region (green), exon (turquoise) or gene + promoter region (grey); b) coding genes and lncRNAs interacting via triplexes in the exon regions, and c) coding genes and lncRNAs interacting via triplexes in the gene (intron and exon) + promoter regions. In b and c, curves are subset according to whether the lncRNA is growth rate associated (DE, purple) or not (NDE, orange). Growth rate association was determined according to a log ratio test between a full or reduced model and an adjusted p-value < 0.0005.
